# 2017 Bailey K. Ashford, Donald Mackay, and Walter Reed Medal Recipients

**DOI:** 10.4269/ajtmh.17-medals

**Published:** 2017-12-06

**Authors:** American Society of Tropical Medicine and Hygiene

Each year, the American Society of Tropical Medicine and Hygiene (ASTMH) honors select individuals with medals recognizing exceptional contributions in tropical medicine and global health. These are the highest honors the Society bestows. They are awarded at the Annual Meeting. ASTMH awarded three Society-level medals and introduced a fourth at the 66th Annual Meeting in Baltimore on November 5, 2017, as follows. The Clara Southmayd Ludlow Medal is described in the preceding article.

## BAILEY K. ASHFORD MEDAL

### The Bailey K. Ashford Medal is awarded for distinguished work in tropical medicine to a worker in his or her early or midcareer.

Margaret Kosek, MD, is the 2017 awardee of the Bailey K. Ashford Medal. Dr. Kosek is an Assistant Professor of International Health, Global Epidemiology, and Control at the Johns Hopkins Bloomberg School of Public Health. Her research objectives are the epidemiology and impact of enteric infections on child health, which continue to be among the principal causes of illness and death in children living in poverty; the improvement of disease burden estimates in child health and a more balanced approach to research priority setting that is directed toward disease burden reduction; and malaria epidemiology, specifically the impact of economic influences and land use patterns on malaria in the Peruvian Amazon.

**Figure:**
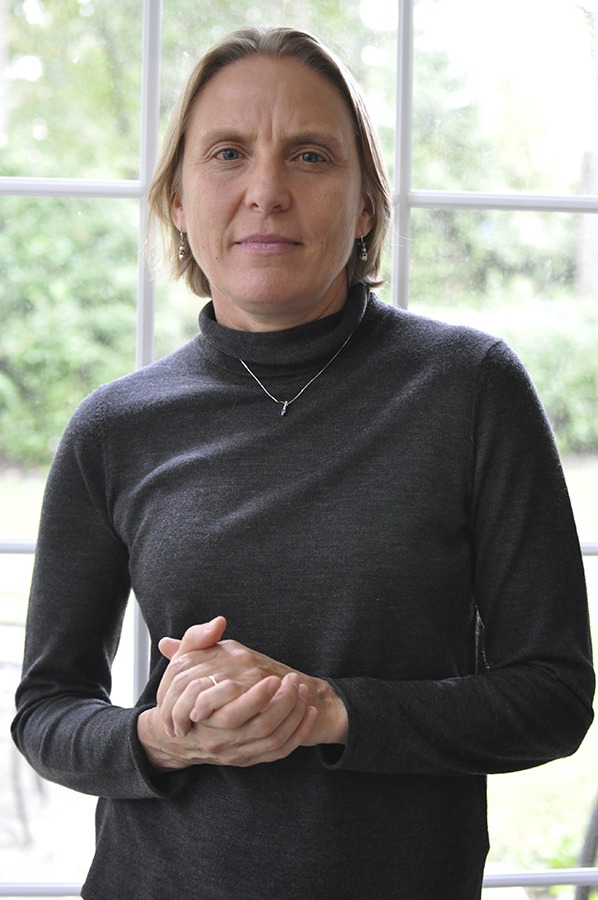
Margaret Kosek, MD, the 2017 Bailey K. Ashford Medal recipient.

**Figure:**
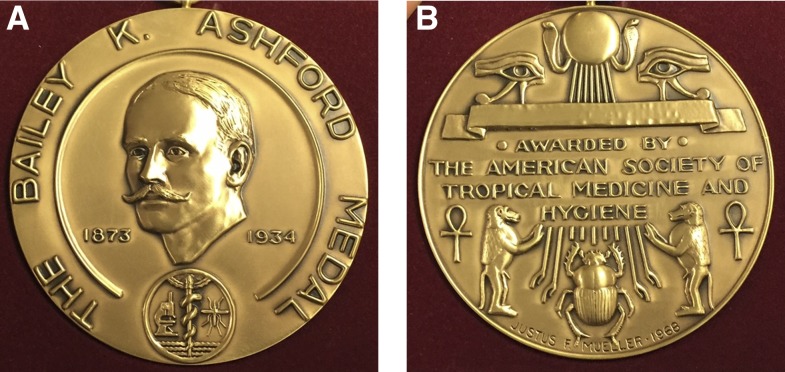
Front (A) and back (B) of the Bailey K. Ashford Medal.

“She captures the spirit of the Ashford medal,” Eric Houpt, MD, FASTMH, wrote in his letter nominating Dr. Kosek. “Bailey Ashford spent most of his professional career in Puerto Rico, and Margaret Kosek has spent most of hers in Peru. She combines that dedication to the field with state-of-the-art biomolecular research to better understand and solve the huge health challenges faced by children and families in impoverished areas in the tropics.”

Dr. Kosek received a Mentored Research Scientist Development Award from the National Institutes of Health in 2001 and she has built, maintained, and staffed a laboratory and field site in Iquitos, Peru, supporting multiple cohort studies and trials concerning childhood illnesses and malaria.

“She carries this remarkably successful research site on her shoulders, a one-woman show,” Dr. Houpt wrote. “… I would be impressed if this were a site led by an entire division, but Dr. Kosek has done this essentially solo, a testament to remarkable leadership and dedication. It does not end with research. While in Iquitos, she still works approximately half of every week as a general practitioner, providing care for free, in the very village where her teams conduct their projects. She has developed remarkable rapport with the community; everyone loves her. It is satisfying and rare to see a person do A to Z, clinical care and research, both laboratory research and clinical research, and she brings it all to the table.”

Dr. Kosek is on the advisory panel of Brighton Collaboration as a member of the diarrhea working group to establish definition and severity scale of diarrhea as an adverse effect of vaccine administration. She is also a reviewer for the National Institute of Allergy and Infectious Diseases Clinical Trial Planning and Implementation grants and a consultant for the Child Health & Nutrition Research Initiative, a Bill & Melinda Gates Foundation funded project to develop an improved system for priority setting in research related to child health. In addition, she is a consultant for the Global Burden of Diseases, Injuries, and Risk Factors Study as a core member in Enteric Infectious Diseases core group.

## DONALD MACKAY MEDAL

### For outstanding work in tropical health, especially relating to improvements in the health of rural or urban workers in the tropics, awarded in odd years by the ASTMH and even years by the Royal Society of Tropical Medicine and Hygiene. Preference is given to medically qualified individuals.

Patrick Lammie, PhD, is the 2017 awardee of the Donald Mackay Medal. Dr. Lammie is the chief scientist for the Neglected Tropical Diseases Support Center (NTD-SC), a program of The Task Force for Global Health. In this role, Dr. Lammie provides technical guidance and strategic oversight into NTD-SC’s operational research projects.

He was formerly a senior staff scientist in the Disease Elimination and Control Group in the Division of Parasitic Diseases and Malaria at the Centers for Disease Control and Prevention (CDC). He worked at CDC for more than 20 years, where his principal focus was lymphatic filariasis. His laboratory was heavily invested in efforts to develop new tools and strategies to monitor and evaluate filariasis and other neglected tropical diseases.

**Figure:**
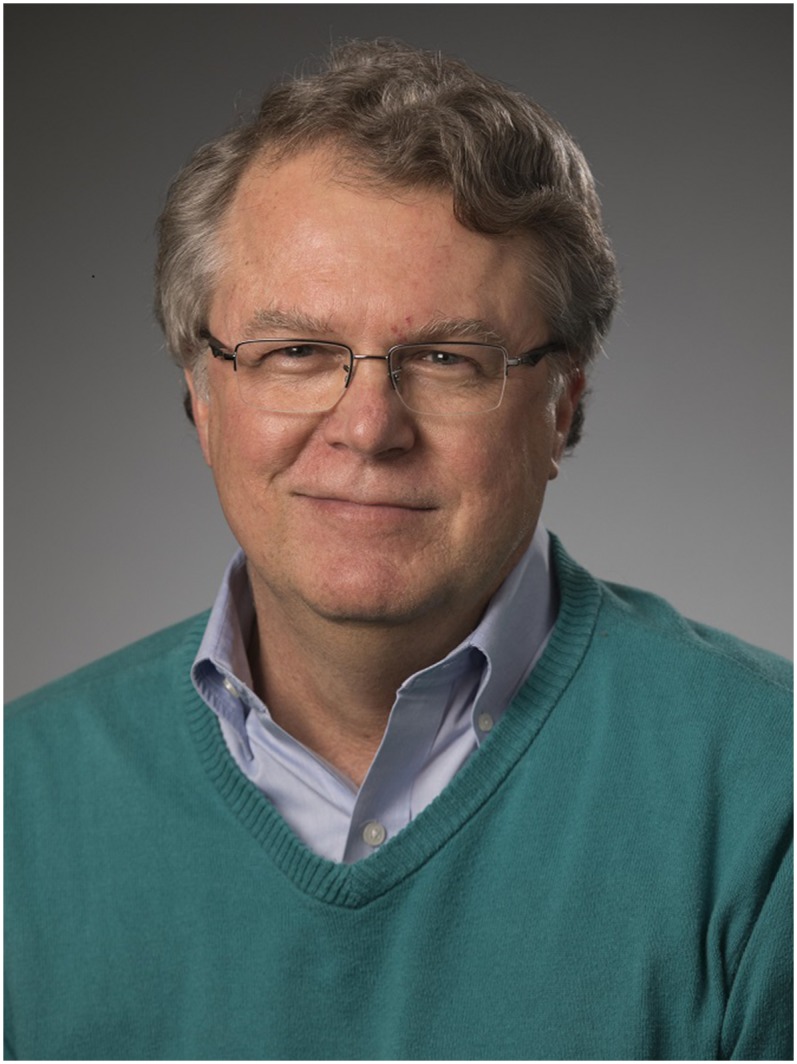
Patrick Lammie, PhD, the 2017 Donald Mackay Medal recipient.

**Figure:**
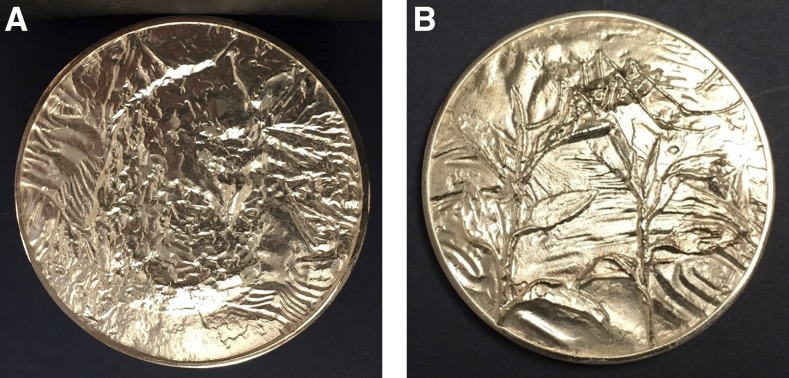
Front (A) and back (B) of the Donald Mackay Medal.

“I can think of no one who is not already a Donald Mackay Medalist who has contributed more to improvements in the health of the underserved in the tropics than Pat Lammie,” Daniel Colley, PhD, FASTMH, wrote in his letter nominating Dr. Lammie. “...For most of his career he has been on the front lines of the elimination of lymphatic filariasis, making it happen. He has provided data needed to implement the programs, implemented the programs, and served as a key leader and chair of countless WHO committees and meetings. Perhaps the most outstanding example of these many activities is his 8 years of effective, consummately competent, highly regarded, and completely trusted cochairing of the Monitoring and Evaluation Committee of the STAG for NTDs at the WHO. This group translates science into practical policy regarding all NTD programs. It moves things forward to benefit people. Through thick and thin he has retained the wide-spread recognition and appreciation of his colleagues and the WHO administration.”

## WALTER REED MEDAL

### The Walter Reed Medal recognizes distinguished accomplishment in the field of tropical medicine.

Scott B. Halstead, MD, FASTMH, is the 2017 recipient of the Walter Reed Medal, the Society’s highest honor. Dr. Halstead has a long and distinguished career in arbovirology and tropical medicine, and he continues to be a strong advocate for arbovirology–especially the development of safe and effective vaccines.

**Figure:**
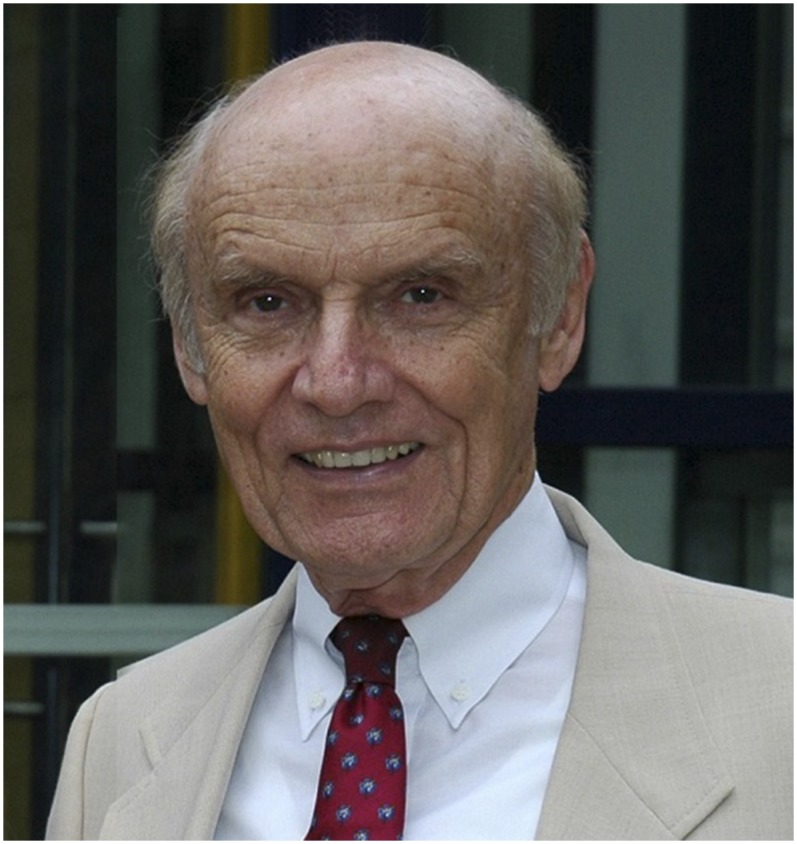
Scott Halstead, MD, FASTMH, the 2017 Walter Reed Medal recipient.

**Figure:**
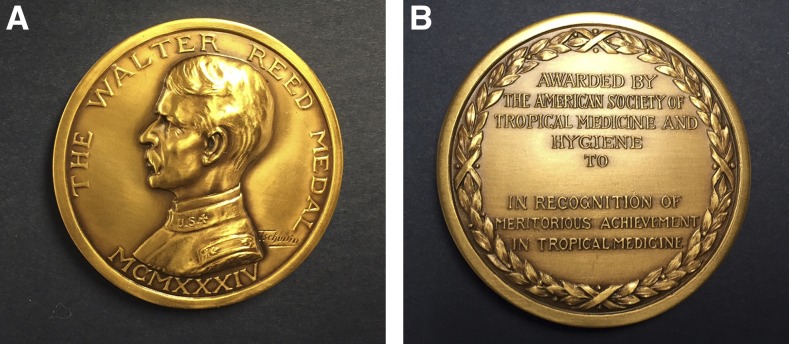
Front (A) and back (B) of the Walter Reed Medal.

“It is my great honor to nominate Dr. Scott Halstead for the Walter Reed Medal in 2017,” Wei-Kung Wang, MD, ScD, wrote in his nominating letter. “Dr. Halstead has made numerous significant contributions to the field of tropical medicine.”

He was the first to recover dengue viruses in tissue culture in 1961 (dengue plaquing) and report the association between secondary dengue infections and increased risk of severe disease. He has devoted much of his scientific career to understanding the molecular mechanisms underlying severe dengue disease. He described antibody-dependent enhancement of dengue virus infection in monkeys in vivo and in human peripheral blood leukocytes in vitro. His hypothesis about antibody enhanced dengue disease is now widely accepted by the scientific community. He has published 293 peer-reviewed articles, 36 invited articles, and 110 book chapters.

In addition, Dr. Halstead contributed importantly to several flavivirus vaccines that are approved or currently being evaluated in clinical trials. He discovered the attenuating properties of the passage of dengue virus in primary dog kidney cells, a discovery that led to the Takeda vaccines currently being evaluated in phase III clinical trials. He promoted the SA 14-14-2 live-attenuated Japanese encephalitis vaccine developed by the Chinese Institute for the Control of Biological and Pharmaceutical Products and played a key role in evaluating the vaccine outside China and in its subsequent wide-spread use in many Asian countries. As the founder and director of the Pediatric Dengue Vaccine Initiative, supported by the Bill & Melinda Gates Foundation and The Rockefeller Foundation, he recruited new investigators to dengue and built a network of researchers across the globe to support the development of dengue vaccines.

“I cannot think of any other living scientist who is more deserving of the Walter Reed Medal than Scott Halstead,” Aravinda de Silva, PhD, wrote in his letter nominating Dr. Halstead. “Dr. Walter Reed’s discoveries about yellow fever and his work with the Yellow Fever Commission in Havana are legendary. Future generations will look back and admire and appreciate the significance of Dr. Halstead’s contributions to arbovirology and tropical medicine.”

Dr. Halstead also founded or led many institutions at the forefront of arbovirology and tropical medicine, including PDVI, the Virology Department of the Armed Forces Research Institute of Medical Sciences in Thailand, and the Department of Tropical Medicine at the University of Hawaii. He was the founder and first chief of the Virology Department, SEATO Medical Research Laboratory (now Armed Forces Research Institute of Medical Sciences) in Bangkok, Thailand, in 1961; founder and president of the International Federation of Tropical Medicine, Antwerp, Belgium, in 1987; and founder and Board Member of the Children’s Vaccine Initiative, Mt. Kisco, NY, in 1991. He is also a past president of the ASTMH.

